# Genomic relationship between polycystic ovary syndrome and bipolar disorder

**DOI:** 10.21203/rs.3.rs-7629869/v1

**Published:** 2025-09-23

**Authors:** Piotr Jaholkowski, Markos Tesfaye, Vera Fominykh, Pravesh Parekh, Erik D Wiström, Nadine Parker, Elise Koch, Anna E. Bauer, Pawel Trzaskoma, Jaroslav Rokicki, Shahram Bahrami, Zillur Rahman, Oleksandr Frei, Srdjan Djurovic, Anders M. Dale, Alexey A. Shadrin, Kevin S. O’Connell, Ole A. Andreassen

**Affiliations:** Center for Precision Psychiatry, Division of Mental Health and Addiction, Oslo University Hospital, and Institute of Clinical Medicine, University of Oslo, Oslo, Norway; Center for Precision Psychiatry, Division of Mental Health and Addiction, Oslo University Hospital, and Institute of Clinical Medicine, University of Oslo, Oslo, Norway

**Keywords:** bipolar disorder, valproate, polycystic ovary syndrome, polycystic ovarian syndrome, comorbidity, genetic

## Abstract

Women with bipolar disorder (BIP) have a higher risk of developing polycystic ovary syndrome (PCOS). Shared genetic architecture may underlie this comorbidity. Valproate, a mood-stabilizer commonly used to treat BIP, increases the risk of PCOS. Still, the mechanism underlying PCOS in BIP remains unknown. Here, we aimed to identify genetic variants shared between BIP and PCOS, as well as their interaction with valproate. We used the results of large-scale genome-wide association studies of BIP (41,510 cases and 354,340 controls), and PCOS (3,609 cases and 229,788 controls). Using conditional false discovery rate, we discovered genetic variants jointly associated with BIP and PCOS. Gene mapping of identified variants was performed using the Open Targets platforms. We analyzed the tissue-specific expression, interaction with valproate, and involvement in biological pathways of the mapped genes. We identified two loci shared between BIP and PCOS. Among the 10 genes mapped to the locus on chromosome 8:11455262, *GATA4, NEIL2*, and *FDFT1* showed expression profiles suggesting their role in the observed comorbidity. Mapped to the locus on chromosome 12:2499849, *CACNA1C, FKBP4, DCP1B*, and *ITFG2* are expressed in both the ovaries and the brain. *CACNA1C* expression is affected by valproate, and *CACNA1C* plays a role in biological pathways involving other valproate-affected genes. We identified shared genetic underpinnings of BIP and PCOS, and implicated genes which may explain the biological mechanisms of the comorbidity between these disorders and a potential mechanism for the role of valproate.

## Introduction

Bipolar disorder (BIP) is a complex mental disorder characterized by recurrent episodes of depression and mania or hypomania (1). A cornerstone in the treatment of BIP is mood-stabilizing drugs, and a commonly prescribed medication is the anti-epileptic drug valproate (VPA) (1, 2). The choice of drug and the course of treatment in BIP are influenced by the fact that both BIP and VPA are independent risk factors for polycystic ovary syndrome (PCOS), characterized by hyperandrogenism, polycystic ovarian morphology, ovarian dysfunction, and subfertility (3–8).

A systematic review and meta-analysis showed a 2-fold higher risk of BIP among women with PCOS (9). This relationship has also been shown in drug-naïve BIP patients, for whom the prevalence of PCOS was estimated at 30% (compared to approximately 10% in the healthy population) (3). Women with BIP, similarly to those diagnosed with PCOS, have higher serum testosterone and androstenedione levels and an increased risk of abnormal menstruation (3, 7, 8, 10, 11). Furthermore, both BIP and PCOS are associated with metabolic imbalances such as hyperinsulinemia, dyslipidemia, metabolic syndrome, and type 2 diabetes (7, 8, 12–14). Although research indicates dysfunction of the hypothalamic-pituitary-adrenal axis, chronic inflammation, altered adipokines, a disturbed gut microbiome, and disturbances in circadian rhythms as potential mechanisms linking BIP and PCOS, the basis of this comorbidity remains elusive (14). This lack of understanding of the mechanism has hindered the development of effective prevention and treatment, thereby increasing the burden of PCOS (7, 8).

Similarly, the mechanism by which VPA increases the risk of PCOS among female patients with BIP, raising its prevalence to over 50%, is unknown (3). Human and animal studies suggest several potential mechanisms: altered gonadotropin-releasing hormone secretion, attenuation of luteinizing hormone (LH) release from the pituitary gland due to enhanced GABAergic inhibitory neurotransmission, direct adenotoxic effects on ovarian tissue, increased ovarian androgen production by theca cells, and metabolic alterations associated with weight gain (3, 15).

Studies have shown that both PCOS and BIP are highly heritable, with heritability estimated at approximately 70% (7, 16–18), and are under polygenic influences (19, 20). Although genetic studies offer important insights into complex diseases and their comorbidity (21–23), our current comprehension of the shared genetic architecture between PCOS and BIP remains limited. Moreover, data-driven genotype phenotype mapping is a valuable resource for identifying drug safety issues, drug repurposing, and the discovery of new drugs (21–25). Therefore, elucidating the shared genetic architecture between BIP and PCOS may contribute to a better understanding of the mechanisms underlying their comorbidity, including those associated with VPA use.

Combining signals from two genome-wide association studies (GWASs) can enhance the detection of shared genetic variants with a second trait for which the GWAS is underpowered (26, 27). The conjunctional False Discovery Rate (conjFDR) approach can identify shared genetic loci between human traits and disorders irrespective of genetic correlation (26). To identify the genetic underpinnings shared between PCOS and BIP, and the potential mechanisms that might underlie PCOS as an adverse event of VPA treatment, we examined the shared genetic architecture using the conjFDR approach (26, 28), followed by extensive functional analyses utilizing available repositories and tools (29–32).

## Materials and methods

### GWAS Samples

The GWAS meta-analysis summary statistics for PCOS (3,609 cases and 229,788 controls) (33) were downloaded from the GWAS Catalog (https://www.ebi.ac.uk/gwas/). The GWAS summary statistics for BIP (41,510 cases and 354,340 controls) were obtained from the BIP Working Group of the Psychiatric Genomics Consortium, after excluding samples from the Estonian Biobank to prevent sample overlap (19). All GWASs included in our analysis were approved by the relevant ethics committees, and informed consent was obtained from all participants.

### Conjunctional FDR

To visualize cross-trait SNP enrichment, we constructed QQ plots that illustrate the distribution of *p*-values for the primary trait conditioned on significance levels in the secondary trait; that is, BIP conditioned on PCOS, and vice versa. To control for spurious enrichment, the QQ plots were generated after random pruning, averaging over 500 iterations. For each iteration of random pruning, all but one random SNP in each linkage disequilibrium (LD)-independent region (clump of SNPs in strong LD, r^2^ > 0.1) were removed, and finally the results were averaged across all iterations. We excluded SNPs within three regions (major histocompatibility complex region chr6: 25119106–33854733; chr8:7200000–12500000; chr19:44909039–45912650) to prevent bias due to complex LD pattern within these regions. Successive leftward deflections on a QQ plot, associated with increasing levels of association with a secondary trait, indicate cross-trait enrichment of a primary trait (26).

We applied the conjFDR approach to identify loci shared between PCOS and BIP (26). The conjFDR method involves conducting two conditional (condFDR) analyses—in our study, conditioning PCOS on BIP and vice versa—which re-rank test statistics and recalculate the associations between variants and a primary trait based on their associations with the secondary trait (26). Then, the conjFDR value for a given genetic variant is defined as the maximum of the two condFDR values, making it a conservative estimate of the association between variants and the traits of interest. In our study, we applied the FDR significance cutoffs at 0.05 for both condFDR and conjFDR. We performed the conjFDR analysis after removing three genomic regions with a complex LD pattern (chr6: 25119106–33854733; chr8:7200000–12500000; chr19:44909039–45912650).

The FUMA protocol was applied to identify independent genomic loci (31). SNPs with conjFDR < 0.05 and an LD r^2^ < 0.6 with each other were defined as independently significant. Lead SNPs were defined as independent significant SNPs with LD r^2^ <0.1 with each other. We defined the boundaries of each genomic locus to include SNPs with an LD r^2^ ≥ 0.6 with any of the independently significant SNPs within the locus. Loci separated by less than 250 kb were merged. A SNP within a given locus that had the lowest conjFDR value was designated as the lead SNP. We applied the 1000 Genomes Project European ancestry haplotype reference panel to compute all LD r^2^ values (34).

### Functional annotation

We applied Open Target Genetics (https://genetics.opentargets.org/) to map genes for lead SNPs from the conjFDR analysis (29) using positional information and an overall score, which is an aggregated measure based on positional information, chromatin interactions, quantitative trait loci, and in silico functional prediction datasets. Adopting a broad inclusion strategy, for each lead SNP we selected the gene with the closest location, along with any genes that had an overall score equal to or higher than that of the closest gene.

We used tissue expression data from the Adult Genotype Tissue Expression Project (GTEx; version 8) to assess the expression of mapped genes in various tissues. These include the brain (cortex, frontal cortex Brodmann area 24, anterior cingulate cortex Brodmann area 24, amygdala, substantia nigra, hippocampus, putamen basal ganglia, caudate basal ganglia, nucleus accumbens basal ganglia, cerebellar hemisphere, cerebellum, hypothalamus, spinal cord cervical C1), pituitary, ovary, subcutaneous adipose tissue, visceral omentum adipose tissue, adrenal gland, cultured fibroblast cells, liver, thyroid, and whole blood. According to the GTEx data presentation approach, isoforms were collapsed into a single gene, and no normalization or threshold procedures were applied. The data used for the analyses were obtained from the GTEx Portal on 26/06/24.

To further explore potential interactions between the mapped genes in the three-dimensional space of the cell nucleus, which could suggest their co-expression (35), we examined published data on chromatin interactions in the brain and ovary (36). We utilized the results of the High-throughput Chromosome Conformation Capture (Hi-C) (37) method to reveal genome-wide 3D chromatin contacts, and visualized interactions using the 3D Genome Browser tool (38). These contacts are essential for understanding the spatial organization of the genome and its impact on gene regulation and cellular function (39). Regions of the genome characterized by a high frequency of interactions compared to other regions are defined as Topologically Associating Domains (TADs). Expression of genes located within a single TAD is more likely to be co-regulated than genes located in separate domains (40).

We used data from the drug–gene interaction database (DGIdb, version 5) to illustrate the strength and type of drug interactions with mapped genes shared between PCOS and BIP (32). Next, we linked the drug-gene interaction data for VPA from DGIdb to data from the Kyoto Encyclopedia of Genes and Genomes (KEGG) (30) to assess the number of pathways encompassing genes affected by VPA and mapped genes. To avoid the double inclusion of CACNA1C as both mapped and affected by the VPA gene, we excluded CACNA1C from the list of genes affected by VPA. We also assessed how many genes affected by VPA were included in pathways that contained the mapped genes. To evaluate whether the number of pathways and genes affected by VPA for each mapped gene exceeded that of a randomly selected protein-coding gene, we compared our estimates to those for 10,000 randomly selected protein-coding genes from a list provided by the GENCODE Project (41). We applied the Bonferroni correction, adjusted for the number of genes mapped to a given locus, to account for multiple testing.

## Results

### QQ plots of SNPs conditioned on association between BIP and PCOS

The conditional QQ plot showed evidence of cross-trait enrichment between BIP and PCOS (Supplementary Fig. 1A). The earlier leftward deflection from the dashed line (no enrichment) indicated a greater proportion of true associations for a given nominal PCOS *p*-value. The more pronounced leftward shifts for decreasing nominal PCOS p-value thresholds demonstrated that the proportion of non-null effects in BIP increased as the strength of the association with PCOS increased. Similarly, the conditional QQ plot displayed polygenic enrichment for PCOS given the strength of association with BIP (Supplementary Fig. 1B).

### ConjFDR analysis

Using condFDR (condFDR < 0.05) we identified 19 distinct genomic loci to be associated with PCOS after conditioning on association with BIP (Supplementary Fig. 2 and Supplementary Table 1). At condFDR < 0.05, we identified 175 loci associated with BIP conditional on PCOS (Supplementary Fig. 3, Supplementary Table 2).

**Figure 1:**
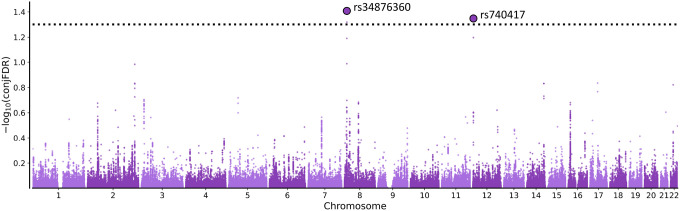
The “ConjFDR Manhattan plots” illustrate SNP FDR values for the joint association between bipolar disorder and polycystic ovary syndrome. The y-axis shows the −log10 transformed conjFDR values. Chromosomal number is presented along the x-axis. The threshold for significant shared associations (conjFDR < 0.05) is represented by the horizontal dotted line. Independent significant SNPs are indicated by a black perimeter.

**Figure 2:**
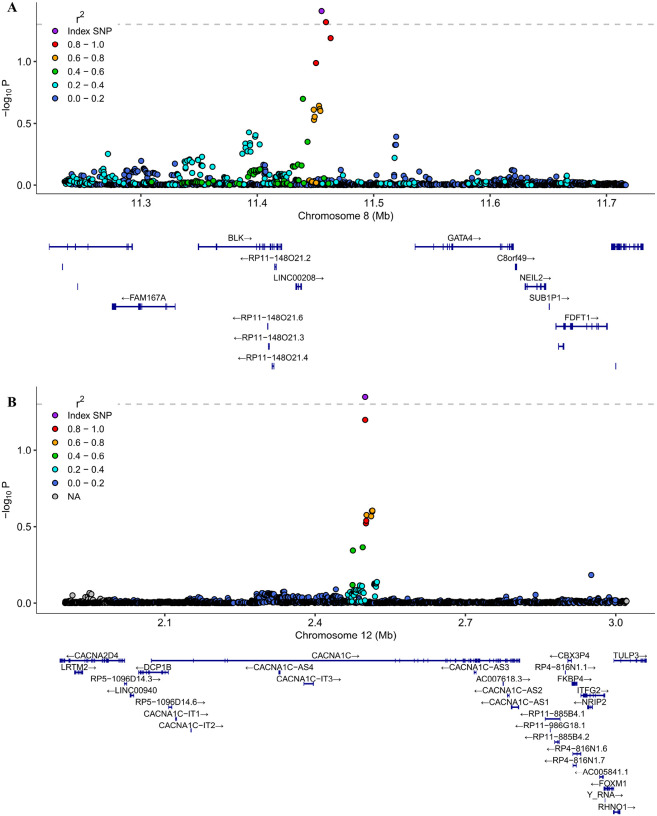
Genetic context for the strongest associations identified in the conjunctional false discovery rate (conjFDR) analysis: (A) rs34876360, (B) rs740417. The y-axis depicts −log10 (conjFDR) values. The dashed horizontal line represents the statistically significant threshold of FDR = 0.05. A single nucleotide polymorphism (SNP) with the strongest association is shown in the large purple circle. The color of the remaining markers reflects the degree of linkage disequilibrium (LD) with the SNP most strongly associated, measured as the r^2^ coefficient and generated from the 1000 Genomes reference data. Genes found within the region are annotated according to *EnsDb.Hsapiens.v75*. The figure is generated with *locuszoomr*.

Applying the conjFDR approach (conjFDR < 0.05), we identified two independent loci jointly associated with PCOS and BIP (Fig. 1C and Fig. 2). One of these loci, with the lead SNP rs34876360, was not identified in the original PCOS GWAS (33) nor in the BIP GWAS (19). The second locus, associated with the lead SNP rs740417, was not identified in the BIP GWAS utilized in our study, nor in the recently published largest BIP GWAS (19, 42).

### Functional analysis

Functional annotation analysis revealed that the lead SNP and 26 SNPs in LD with the lead SNP for the locus shared between PCOS and BIP on chromosome 8:11455262, displayed an intergenic character. The lead SNP and 10 SNPs in LD with the lead SNP for the locus identified on chromosome 12:2499849 were recognized as intronic of *CACNA1C* (Fig. 2).

**Figure 3:**
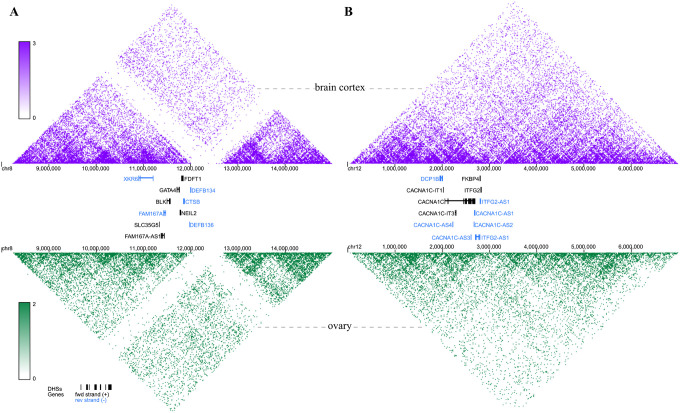
Chromatin interactions (Hi-C) for regions surrounding: (A) locus located on chromosome 8:11455262 (rs34876360), (B) locus on chromosome 12:2499849 (rs740414) shared between bipolar disorder and polycystic ovary syndrome. Top (purple) panels illustrate Hi-C in brain cortex. Bottom (green) panels illustrate Hi-C in ovary tissue. Between the Hi-C panels, the locations of genes mapped to the identified loci are shown. For clarity, the locations of the remaining genes are omitted. The intensity of the colors illustrates the strength of interactions between pairs of loci and regulatory elements. Results are displayed with 25kb resolution in the hg38 genome reference assemble.

The overall Open Targets variant-to-gene analysis for the lead SNP of the locus shared between PCOS and BIP and located on chromosome 8:11455262 (rs34876360) revealed 9 genes (*BLK, SLC35G5, XKR6, DEFB134, FDFT1, DEFB136, FAM167A, NEIL2, CTSB*) in addition to the nearest *GATA4*. Similar analysis for the lead SNP on chromosome 12:2499849 (rs740417) identified *CACNA1C* (an intronic variant), *FKBP4, ITFG2*, and *DCP1B* as the nearest gene. Tissue-specific gene expression (GTEx) analysis for genes mapped to the first locus showed that *FDFT1* and *NEIL2* were expressed at moderate level in both the brain and ovary (Fig. 3). Other genes (*BLK, CTSB, DEFB134, DEFB136, FAM167A, SLC35G5, XKR6*) were either not detected or expressed at low levels in both the brain and ovary, or in either organ individually (Supplementary Fig. 4). Interestingly, GATA4 was specifically and highly expressed in ovarian tissue but not expressed in the brain (Fig. 3). For the second locus, we observed expression of CACNA1C and *FKBP4* within a similar range in both the ovary and most brain tissues, while *DCP1B* and *ITFG2* tended to have higher expression levels in the ovary compared to the brain (Supplementary Fig. 5).

Our analysis revealed that regions on chromosome 8:11455262 (Fig. 3A) and chromosome 12:2499849 (Fig. 3B), which encompass the identified genes of interest, are enriched for chromatin contacts in both the brain cortex (upper panel) and ovary (lower panel).

The DGIdb analysis revealed that *CACNA1C* is one of the genes affected by VPA (Supplementary Fig. 4–6). Furthermore, it revealed that other therapeutic substances are known to block or modulate the expression of *CACNA1C* (Supplementary Fig. 4).

**Figure 4:**
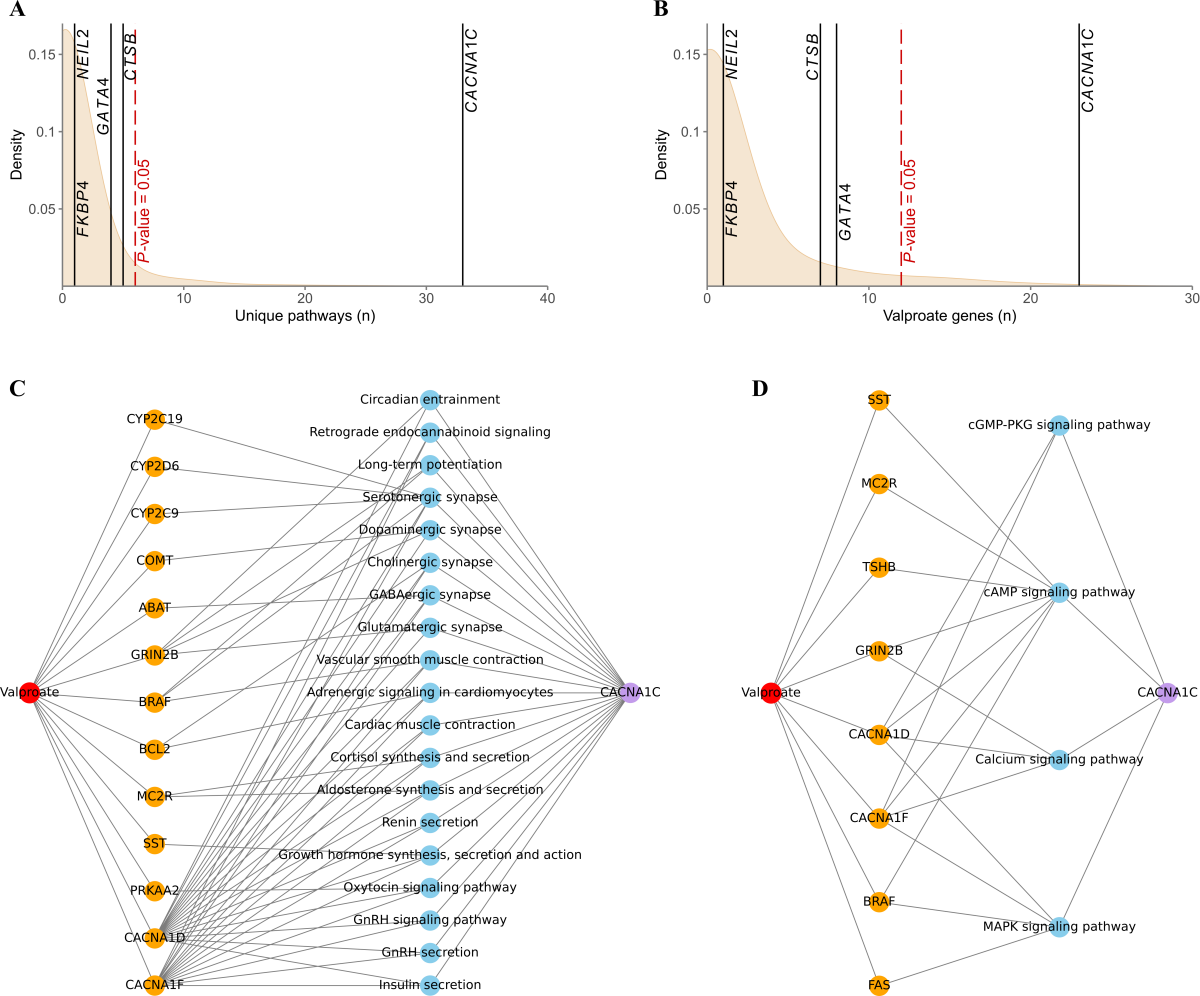
The biological pathways encompass both the gene(s) affected by valproic acid (VPA) and the genes mapped for loci shared between bipolar disorder (BIP) and polycystic ovary syndrome (PCOS). (A) The number of unique biological pathways encompassing gene(s) affected by VPA and individual genes is depicted. The density distribution of the number of unique pathways containing genes regulated by VPA, along with 10,000 individual genes randomly selected from protein-coding genes, is shown in beige. The red dashed line represents the nominal p-value for the given density distribution. Black lines represent the number of unique genes encompassing VPA-regulated gene(s) and a given gene mapped to loci shared between BIP and PCOS. (B) The number of unique gene(s) affected by VPA that are included in biological pathways also encompassing the gene of interest. Details are analogous to those described for the plot in (A). (C) The network illustrating organismal systems pathways (blue) encompassing *CACNA1C* and VPA-modified genes (orange). (D) The network depicts environmental information processing pathways encompassing *CACNA1Cand* genes modified by VPA. To enable the above analyses, we removed *CACNA1Cfrom* the list of genes affected by VPA.

In our analysis of KEGG-annotated pathways containing genes affected by VPA, as well as those mapped to loci jointly associated with PCOS and BIP, we identified 33 pathways associated with *CANCA1C*, five with *CTSB*, four with *GATA4*, and one each involving *NEIL2* and *FKBP4* (Fig. 4A). This analysis further revealed that *CACNA1C* shares biological pathways with 23 other genes, *GATA4* with eight, *CTSB* with seven, and both *NEIL2* and *FKBP2* with one gene influenced by VPA (Fig. 4B). We observed that for *CACNA1C*, both the number of biological pathways and the number of genes affected by VPA were statistically higher than those observed for randomly selected protein-coding genes (*p*-values = 0.019 and 0.032, respectively).

## Discussion

In our study, we applied the conjFDR framework to genome-wide data to identify the genetic underpinnings of BIP and PCOS comorbidity. We identified two independent loci jointly associated with BIP and PCOS and mapped several genes to them, such as *GATA4, NEIL2*, and CACNA1C, highlighting the potential role of these gene in disorder comorbidity. Furthermore, the results indicate that the mapped genes may be involved in the development of PCOS associated with VPA use. This provides new insight into the molecular pathways involved in PCOS and BIP comorbidity, as well as PCOS associated with VPA, which can form the basis of experimental work to determine the mechanisms and form the basis of future drug development to avoid these adverse events.

We observed cross-trait polygenic enrichment between PCOS and BIP, supporting previous epidemiological observations of comorbidities (3, 9). The two overlapping loci between PCOS and BIP exhibited both concordant and discordant directions, similar to those observed in many other pairs of human traits (43–45).

The functional analysis included gene mapping of identified shared loci, and we utilized broad inclusion criteria based on OpenTargets to minimize the risk of omitting genes related to the traits of interest. For the loci on chromosome 8:11455262, apart from the nearest *GATA4*, we identified nine genes (*BLK, SLC35G5, XKR6, DEFB136, FDFT1, DEFB134, FAM167A, NEIL2, CTSB*). Importantly, although GATA4 was not identified in the original GWAS for PCOS that we utilized for the conjFDR analysis, *GATA4/NEIL2* locus (rs804279; chr8:11623889) was identified in an independent GWAS for PCOS (46). This finding confirms the involvement of this genetic locus in the pathogenesis of PCOS and validates the conjFDR methodology used in our study. The lead SNP (rs740417) within the locus shared between PCOS and BIP on chromosome 12:2499849 was identified as an intronic variant of *CACNA1C*, and located in proximity to *FKBP4, ITFG2*, and *DCP1B* genes.

The results of tissue-specific gene expression analysis for genes mapped to the locus on chromosome 8:11455262 showed that *GATA4* is highly specific to the ovary, while other mapper genes (i.e., *FDFT1* and *NEIL2* = are expressed in both the brain and the ovary. These findings provide further evidence for their possible involvement in the comorbidity of PCOS and BIP. For the locus on chromosome 12, tissue expression analysis indicates that *CACNA1C, FKBP4, DCP1B*, and *ITFG2* are all expressed in both the ovary and the brain, with a noteworthy similarity in the expression patterns of *DCP1B* and *ITFG2*. Moreover, the results of the Hi-C analysis demonstrated that, for both loci, the mapped genes are not only closely located in terms of genomic distance but also maintain physical proximity within nuclear space, suggesting that they may be co-expressed. This implies that genetic variants or modulators of the mapped genes such as VPA, could influence the transcription of multiple genes, with several genes potentially contributing to the comorbidity of PCOS and BIP.

The DGIdb analysis revealed that VPA affects *CACNA1C*. Additionally, we observed that the genes *FKBP4, NEIL2, GATA4, CTSB*, and *CACNA1C* participate in common biological pathways with those affected by VPA. This is particularly relevant for *CACNA1C*, which is involved in biological pathways such as MAPK signaling and calcium signaling that may explain the observed comorbidity (47–50), as well as underlie the metabolic disorders observed in both PCOS and BIP (51, 52). *GATA4*, the nearest gene to the lead SNP (rs34876360) in the locus shared between PCOS and BIP on chromosome 8:11455262, is a zinc-finger transcription factor. It is a transcriptional inducer of p15INK4B, a cyclin-dependent kinase inhibitor, leading to the reduction of cyclin D1 (53). *GATA4* is critical in organogenesis, particularly in the development of the heart and gonads (54, 55). Moreover, animal studies have shown that *GATA4* plays an important role in the development of GnRH neurons and GnRH gene expression (56, 57). Combined with the observed dysfunctions of the hypothalamic-pituitary-gonadal axis in PCOS and the crucial role of GnRH analogues in its treatment, these findings further suggest that *GATA4* may play a role in the pathogenesis of PCOS (7, 8). It is expressed in the developing and adult brain and negatively regulates the proliferation and growth of astrocytes (53). Interestingly, VPA exerts the opposite effect of stimulation of astrocyte proliferation in the developing brain (57).

The co-occurrence of cardiovascular disease in BIP patients (58, 59), and the association of PCOS with major adverse cardiovascular events from a young age, independent of body-mass index (60, 61), further underscores the significance of *GATA4* as a potential unifying factor. *NEIL2* encodes a DNA glycosylase that initiates base excision DNA repair by cleaving oxidatively damaged bases, and it is crucial for long-term genomic maintenance (62). *NEIL2* has been linked with PCOS by the previous GWAS (63). It has been shown that mice lacking Neil2 display hyperactivity and reduced anxiety, endophenotypes typical of animal models of BIP (64, 65). Moreover, *Neil2* knock-out mice were associated with reduced reactivity of NR2A subunits of NMDA receptors, which have been linked with the BIP pathogenesis (66). Noteworthy, altered *Nr2a* expression has been observed in VPA-exposed rats (67). *CTSB* encodes cathepsin B, a lysosomal protease and has been linked with pathogenesis of PCOS (68). Previous study based on microarray-based gene expression profiling indicated that *Ctsb* effects on depression-like behavior in mice (69). Moreover, it has been demonstrated in clinical settings that VPA increases cellular level of cathepsin B (70). *FDFT1* encodes farnesyl-diphosphate farnesyltransferase 1, an evolutionarily conservative enzyme involved in the cholesterol biosynthesis (71, 72). It has been demonstrated in an animal study that *Fdft1* is involved in follicular development in ovaries (72). The recessive variants of *FDFT1* have been linked with profound developmental delay, brain abnormality, irritability, and sleep disturbances (71).

*CACNA1C* encodes calcium voltage-gated channel subunit alpha1 C. Multiple genetic studies, including GWAS, have linked *CACNA1C* with BIP (19, 73, 74). Increased serum levels of *CACNA1C* in BIP has been recently reported (75). *CACNA1C*, apart from its involvement in processes such as neurotransmitter release and synaptic plasticity (76), also affects the functioning of mitochondria and lysosomes (80), which are linked to the pathogenesis of PCOS (77). *FKBP4* encodes FKBP prolyl isomerase 4 (FKBP4), which is involved in immunoregulation and cellular trafficking of steroid hormone receptors (78). Higher expression of *Fkbp4* has been observed in a rat model of PCOS (78). Human chorionic gonadotropin stimulation upregulates expression of *FKBP4* in human ovulatory follicles (79). Moreover, FKBP4 was shown to be involved in the intranuclear translocation of the glucocorticoid receptor (NR3C1) which was in turn linked to the pathogenesis of PCOS (79–82). Increased expression of *Fkbp4* has been observed in the hypothalamus of a rat model of depression (83). Genetic association analysis showed a relationship between *FKBP4* polymorphism and stressful life events in patients with BIP (83). Furthermore, decreased *FKBP4* mRNA level has been observed in schizophrenia (84).

The observations of the mapped genes may provide a basis for further targeted analyses in animal models (85) and clinical conditions. It is particularly important to evaluate the utility of identifying genetic variations within mapped genes, especially *CACNA1C*, to assess the risk of developing PCOS prior to initiating VPA treatment. However, it should be emphasized that the results indicating potential co-expression of several genes within the identified locus, along with biological pathways connecting *CACNA1C* to genes modified by VPA, highlight the complexity of the observed processes. This complexity advises caution against adopting overly simplified experimental assumptions.

The main limitation of our study is that we used only summary statistics from GWASs of individuals with European ancestry, which limits the generalization of the results. Due to the absence of a large-scale female-specific GWAS for BIP, we conducted our analysis using data from both sexes. However, the genetic loci we identified show consistent effect direction and nominal significance in available female-specific GWAS. Further, our functional analysis is based on associations and should be verified by experimental studies.

## Conclusions

Here, we identified genetic variants shared between BIP and PCOS, and functional analyses implicated genes representing molecular pathways underlying the comorbidity between PCOS and BIP. The identified pathways were also linked to the pharmacological mechanisms of VPA. These findings provide new understanding of the pathophysiology of PCOS associated with BIP and VPA use.

## Supplementary Material

Supplementary Files

This is a list of supplementary files associated with this preprint. Click to download.

- Genomicrelationshipbetweenpolycysticsuppmat16092025.docx
- Genomicrelationshipbetweenpolycysticsuppltabl16092025.xlsx
